# Synthetic Iron Oxides for Adsorptive Removal of Arsenic

**DOI:** 10.1007/s11270-018-3866-2

**Published:** 2018-06-08

**Authors:** Izabela Polowczyk, Piotr Cyganowski, Justyna Ulatowska, Wojciech Sawiński, Anna Bastrzyk

**Affiliations:** 1 Division of Chemical Engineering, Wroclaw University of Science and Technology, Wybrzeże S. Wyspiańskiego 27, 50-370 Wrocław, Poland; 2 Division of Polymer and Carbonaceous Materials, Faculty of Chemistry, Wroclaw University of Science and Technology, Wybrzeże S. Wyspiańskiego 27, 50-370 Wrocław, Poland

**Keywords:** Akaganeite, Hexadecyltrimethylammonium bromide, Adsorption, Kinetics, Iron oxide, Pollution, Arsenic contamination

## Abstract

Removal of arsenic from water reservoirs is the issue of great concern in many places around the globe. As adsorption is one of the most efficient techniques for treatment of As-containing media, thus the present study concerns application of iron oxides-hydroxides (akaganeite) as adsorbents for removal of this harmful metal from aqueous solution. Two types of akaganeite were tested: synthetic one (A) and the same modified using hexadecyltrimethylammonium bromide (A_M_). Removal of As was tested in batch studies in function of pH, adsorbent dosage, contact time, and initial arsenic concentration. The adsorption isotherms obey Langmuir mathematical model. Adsorption kinetics complies with pseudo-second-order kinetic model, and the constant rates were defined as 2.07 × 10^−3^and 0.92 × 10^−3^ g mg^−1^ min^−1^ for the samples (A) and (A_M_), respectively. The difference was caused by significant decrease in adsorption rate in initial state of the process carried out for the sample A_M_. The maximum adsorption capacity achieved for (A) and (A_M_) akaganeite taken from Langmuir isotherm was 148.7 and 170.9 mg g^−1^, respectively. The results suggest that iron oxides-hydroxides can be used for As removal from aqueous solutions.

## Introduction

Arsenic is one of the most hazardous pollutants in many regions of the world as it can be easily solubilized in groundwater (Giri and Patel 2011). The problem concerns USA, China, Chile, Bangladesh, Taiwan, Mexico, Argentina, Poland, Canada, Hungary, New Zealand, Japan, and India (Mohan and Pittman 2007). Arsenic contamination is usually linked with highly developed industry (mining and metallurgy), geothermic processes, volcanic eruptions, and biological activity (Smedley and Kinniburgh 2002). Arsenic in the water environment occurs both organic as well as inorganic forms. This mainly includes As oxyanions: arsenite (H_2_AsO_3_^−^) and arsenate (H_2_AsO_4_^−^) (Sarı and Tuzen 2009; Mohan et al. 2007). Arsenic (III) is the most toxic and mobile in the environment. It has been reported that the arsenic poisoning causes melanosis; edema; keratosis; dark spots on the chest; enlargement of liver, kidney, and spleen; and cancers of skin, lungs, and urinary bladder (Giri et al. 2011; Wang and Mulligan 2008; Choong et al. 2007). For this reason, the World Health Organization (WHO) has recommended the maximum concentration of arsenic in drinking water as 10 μg L^−1^ (Mohan and Pittman 2007). However, it was observed that the typical concentration of this metal in contaminated water used for human consumption is about 100–300 μg L^−1^ (Zaw and Emett 2002).

Removal of arsenic(III) ions from water can be achieved by various techniques, such as coagulation (Song et al. 2006; Wickramasinghe et al. 2004) and reverse osmosis (Ning 2002). Most of these methods suffer from high operational cost and incomplete metal removal (Giri et al. 2011; Sharma and Sohn 2009). In contrast, adsorption is an effective method (Sarı and Tuzen 2009; Mohan and Pittman 2007; Kundu and Gupta 2007; Pirilä et al. 2011) and can be considered as economically justified. Studies on adsorption of arsenic(III) were carried out on various adsorbents, including fly ashes (Polowczyk et al. 2010; Cho et al. 2005), activated carbon (Pattanayak et al. 2000), natural and synthetic clay materials (Guo et al. 2011; Liao et al. 2011), ion-exchange resins (Pakzadeh and Batista 2011; Kim and Benjamin 2004), multi-walled carbon nanotubes (V.K. Gupta and Saleh 2013; Saleh et al. 2011), and metal oxides (Pirilä et al. 2011; Deliyanni et al. 2006; Mostafa et al. 2011). However, charge of As(III) is neutral in the media of concern (utility water); thus, it is difficult to remove. For this reason, a potential method for treatment of As-containing wastes must consider pH of the environment to provide oxidation of As(III) to As(V) which species reveal ionic character (Yazdi and Darban).

Iron oxides have been widely used as adsorbents for removal of various contaminants from water, wastewater, and liquid hazardous wastes (Deliyanni et al. 2003; V. K. Gupta and Nayak 2012). Their use is limited due to the competition with the commercially available natural aluminum oxides (Anderson et al. 1976), biomass (Sarı and Tuzen 2009), biogenic schwertmannite (Liao et al. 2011), soot (Pattanayak et al. 2000), and fly ashes (Cho et al. 2005; Aguilar-Carrillo et al. 2006; Polowczyk et al. 2010). Ferric oxides and hydroxides are available only as fine powders or are generated in aqueous suspensions as a hydroxide floc or gel. In these forms, they retain their desirable sorptive properties for various elements (Deliyanni et al. 2006).

One of the type of ferric products is akaganeite (β-FeO(OH)). Akaganeite occurs in nature in hot brines of Atlantis Deep of the Red Sea and the hot springs of similar composition of the White Island volcano, New Zealand (Schwertmann and Cornell 2007). This mineral is formed in nature under the same conditions as these used in laboratory synthesis, i.e., in the presence of FeCl_3_ at elevated temperatures. Advantages of this adsorbent result from its nano-structured character, leading to high surface area and narrow pore size distribution (Deliyanni et al. 2003; Deliyanni et al. 2006; Deliyanni and Matis 2005). Akaganeite-based adsorbents have been successfully applied in recent years to remove inorganic arsenic species from drinking water (Kolbe et al. 2011). As the starting reagents for its synthesis are cheap and readily available, a potential application of akaganeite as an adsorbent can be recognized as economically efficient (Deliyanni et al. 2003). The mineral adsorbent can be modified by introducing onto its surface compounds revealing ionic character. For instance, hexadecyltrimethylammonium bromide (HDTMA) can modify chemically the surface of akaganeite at relatively small concentrations, as ionic surfactants are considered in general to sorb onto the surfaces of charged adsorbent by an ion exchange mechanism. The available data describing such materials provide the information about their suitability for the removal of As(III) species (Deliyanni et al. 2006). However, if the As(III) may be easily oxidized to ionic species of As(V), introduction of a surfactant can enhance adsorption of the so-prepared materials, increasing a role of ion exchange reactions acting during the process.

For this reason, the overall objective of this study was to determine whether surfactant modification of akaganeite is sufficient for enhancing adsorption of arsenic on such materials. The studies were carried out simultaneously for ferric oxide, pure akaganeite (β-FeO(OH)) (A), and its surfactant-modified derivative (A_M_). Within the present studies, total adsorption capacity, adsorption equilibrium, and adsorption kinetics were determined for these two adsorbents. The carried out analyses allowed to answer to questions: (1) Is the modification of hydrous ferric oxide with the HDTMA efficient? (2) Is such an approach enhancing adsorption of arsenic?

## Materials and Methods

### Materials

HDTMA was acquired from Alfa Aesar. The arsenic as well as FeCl_3_ and NaOH were purchased in Avantor Performance Materials Ltd. (Poland). All of the reagents were used as received, without pre-treatment.

### Preparation of Akaganeite Adsorbent

The preparation of akaganeite was carried out according to the method previously presented in the reference cited under Chitrakar et al. (2006). Two types of adsorbents were obtained: (A) akaganeite and (A_M_) akaganeite modified with HDTMA.

The sample A was obtained as follows: a 2-L beaker containing 60 mL of 0.1 mol L^−1^ FeCl_3_ solution was placed on a magnetic stirrer. Then, 0.1 mol L^−1^ NaOH was slowly added, until the pH of the mixture reached a value of 10. After this, the content of the beaker was mixed for additional 1 h; then, the so-prepared suspension was inserted in a cellulose dialysis membrane for 7 days to remove chlorine anions.

The sample A_M_ was obtained applying the same procedure as described above with the difference that additionally 6 mL of 0.1 mol L^−1^ HDTMA solution was added drop-by-drop to the reacting mixture. The reaction product was then placed in a cellulose dialysis membrane for 7 days to remove chlorine anions.

### Methods

Analysis of particle size was carried out using a Mastersizer 2000 laser diffractometer, equipped with HydroMu dispersion unit (Malvern Instruments). The specific surface areas of the akaganeite (A) and the modified akaganeite (A_M_) were determined by tests on sorption and desorption of helium/nitrogen mixture using FlowSorb 2300 apparatus (Micromeritics). The received data were then recalculated using Brunauer-Emmett-Teller (BET) equation. The scanning electron microscope (SEM) micrographs were captured on a S-3400N microscope (HITACHI).

Concentration of As was analyzed spectrophotometrically using UV-Visible spectrophotometer Evolution 201 (Thermo Scientific) by means of the molybdenum blue method, according to appropriate standard procedure. Then, the adsorption (mg g^−1^) and arsenic removal (%) were calculated from the initial and equilibrium concentrations determined after 24 h of the process (Giri et al. 2011).

Adsorption kinetics was modeled using a pseudo-second-order (PSO) and intraparticle pore diffusion (IPD) kinetic models in their linear forms (Ho and McKay 1999; Shi et al. 2009; Ho et al. 2000), respectively:

1 $$ \frac{t}{q_t}=\frac{1}{k_2{q}_m^2}+\frac{t}{q_m} $$

2 $$ {q}_t={k}_d{t}^{\raisebox{1ex}{$1$}\!\left/ \!\raisebox{-1ex}{$2$}\right.}+\theta $$

where *k*_2_ is the PSO rate constant (g mg^−1^ min^−1^), *k*_*d*_ is the IPD rate constant (mg (g^−1^ min^−1/2^)), *q*_*m*_ is the adsorption capacity at equilibrium (mg g^−1^), and *q*_*t*_ is the amount of sorbate on the surface of the sorbent at any time *t* (mg g^−1^). Θ (mg g^−1^) is a factor related to the thickness of the boundary layer; this value is proportional to boundary layer effect.

Equilibrium studies were carried out by fitting the experimental data to the linearized Langmuir equation:

3 $$ \frac{c_{eq}}{q_e}=\frac{1}{Q_0b}+\frac{c_{eq}}{Q_0} $$

The separation factor (dimensionless) called equilibrium or separation parameter (*R*_*L*_) was calculated from the equation:

4 $$ {R}_L=\frac{1}{1+{bc}_0} $$

where *c*_0_ is the initial concentration of As (mg L^−1^), *c*_eq_ is the equilibrium concentration (mg L^−1^), *q*_*e*_ is the experimental adsorption capacity at equilibrium (mg g^−1^), *Q*_0_ is the adsorption capacity (mg g^−1^), and *b* is the Langmuir adsorption constant related to the adsorption energy (L mg^−1^).

### Adsorption of As

Arsenic trioxide (As_2_O_3_) was used as the source of As ions. A stock solution (1000 mg L^−1^) was prepared in deionized water in the presence of NaOH. The so-prepared solution was acidified with 2.0 mol L^−1^ HCl (pH 3) and diluted to 1 L with deionized water.

All the adsorption studies were carried out using batch method at room temperature and pH 7 for 24 h. The effect of pH was investigated by adjusting the pH of a set of As solutions using 0.1 M HCl and 0.1 M NaOH, respectively. Then, 11 mg of an adsorbent (A or A_M_) per each 10 mL of a solution was taken. The effect of adsorbent dose was studied by varying the dose of an adsorbent (A or A_M_) from 1.1 to 55 mg for initial As concentration of 100 mg L^−1^. The equilibrium studies of As adsorption were carried out using 11 mg of an adsorbent, introduced into 10 mL of As solutions that concentration was varied in range of 20–250 mg L^−1^. The kinetic experiments were conducted in batch mode by shaking 11 mg of an adsorbent with As solution at a constant pH (~7). As concentration was monitored for 3 h, starting from third minute of the process.

## Results and Discussion

### Characterization of Adsorbents

In Table 1, the particle size analysis of pure akaganeite showed volume median diameter (*d*_50_) of 22.0 μm, while the same parameter determined for the modified akaganeite was 28.7 μm. The corresponding values of *d*_10_ and *d*_90_ were also higher in the case of the sample A_M_ (Table 1). The phenomenon must had been caused by the effect of surfactant (HDTMA) addition to the mixture which the adsorbent (A_M_) was precipitated from. The presence of the surface-active agent simply allowed bigger agglomerates to be obtained. Substantial differences can be observed in the specific surface areas of both samples. They are, respectively, 254.0 (A) and 26.0 m^2^ g (A_M_). Such a ten-fold difference may indicate that the HDTMA filled up the pores of the akaganeite; thus, the modification might indeed occur.

**Table 1:** Characteristics of the obtained samples

Sample	*d*_50_^a^	*d*_10_^a^	*d*_90_^a^	*S*_BET_^b^
A	22.02	8.35	48.34	254
A_M_	28.72	10.02	62.34	26

*A* pure akaganeite, *A*_*M*_ modified akaganeite

^a^Diameter percentile (μm)

^b^Specific surface area (m^2^ g^−1^)

**Fig. 1:**
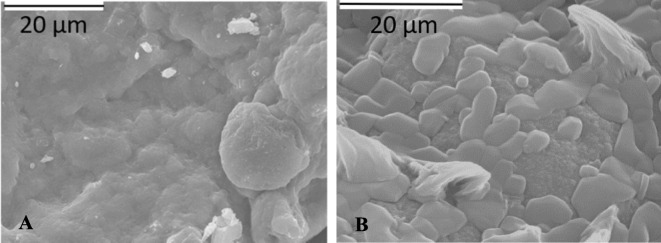
SEM micrographs of **a** akaganeite and **b** akaganeite modified with HDTMA

Morphology of the obtained adsorbents was investigated by scanning electron microscopy (SEM). The captured micrographs are displayed in Fig. 1.

As can be seen in Fig. 1, unmodified akaganeite is characterized by uniform compact structure, while on the surface of the HDTMA-modified one supports longitudinal particles, previously recognized as an indicator of mineral modification using surfactant (Deliyanni et al. 2006).

### Effect of Adsorbent Dosage

**Fig. 2:**
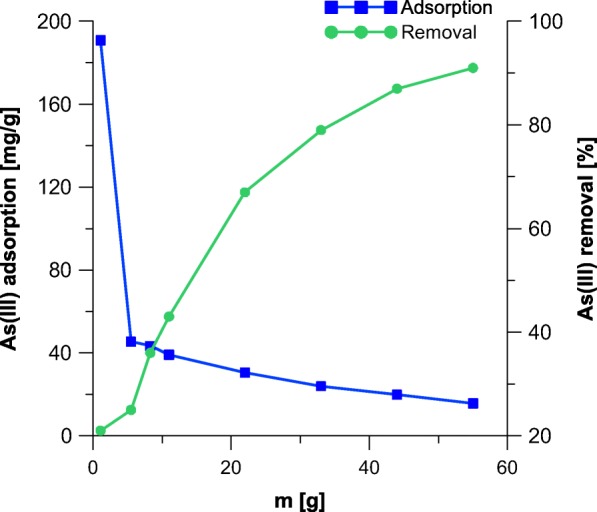
The adsorption capacity of akaganeite towards As

**Fig. 3:**
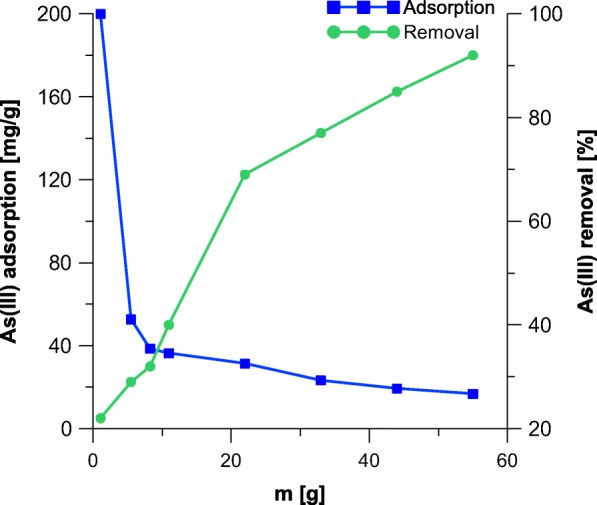
The adsorption capacity of modified akaganeite and As removal

Figures 2 and 3 display the effect of adsorbent dose on the removal of As. The maximum static uptake of As by the samples A and A_M_ was achieved for minimal possible adsorbent-to-solute ratio of 0.11 mg/mL and were 190.9 and 198.1 mg g^−1^, respectively. The maximum removal of As was achieved for maximal dose of adsorbent (above 90%). The obtained results are interesting, as already reported in the literature maximum values of adsorption for different ions on akaganeite are for instance 0.65 mg g^−1^ of antimony (Kolbe et al. 2011), 10 mg g^−1^ of phosphates (Chitrakar et al. 2006), 80 mg g^−1^ of chromium (VI) (Lazaridis et al. 2005), and 17.1 mg g^−1^ of cadmium (Deliyanni and Matis 2005).

### Effect of pH

**Fig. 4:**
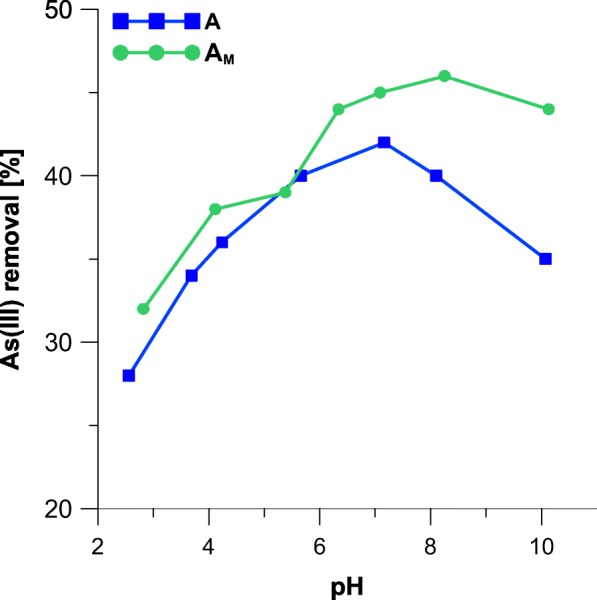
Effect of solution pH on As adsorption onto akaganeite (A) and modified akaganeite (A_M_)

The samples A and A_M_ were contacted with solutions that pH has varied in range from 2.4 to 10. Figure 4 displays effect of pH on the adsorption of As. It was observed that As adsorption increases with the increase of solution pH to about 8. However, the adsorption of As decreases slightly when solution pH > 8.5. The maximum removal of As occurs at pH 7–8.5, in which the removal percentage was more than 45% for the sample A_M_. The sample A_M_ reveals an overall greater sorption capacity that the unmodified mineral, except of pH 5.4, where adsorption capacity is almost equal for both samples. The effect must be attributed to the presence of quaternary ammonium groups in the sample A_M_. In both acidic or basic pH, the functionalities are bearing exchangeable ions (Cl^−^ or OH^−^), while at neutral pH, it exists in form of free amine. This further can be linked with speciation of As, as the metal exists in ionic forms at pH between 7 and 8 (Takeno 2005). For this reason, it can be assumed that adsorption on the sample A_M_ may employ an ion-exchange mechanism. This could also explain why the removal efficiency is not dependent on specific surface area (which is ten times higher in case of the sample A). Hence, the adsorption of As on the modified iron oxide may reveal a chemical character. For this reason, the further adsorption studies were carried out at natural pH (7) of the solutions (see Sect. 2).

### Adsorption Kinetics

**Fig. 5:**
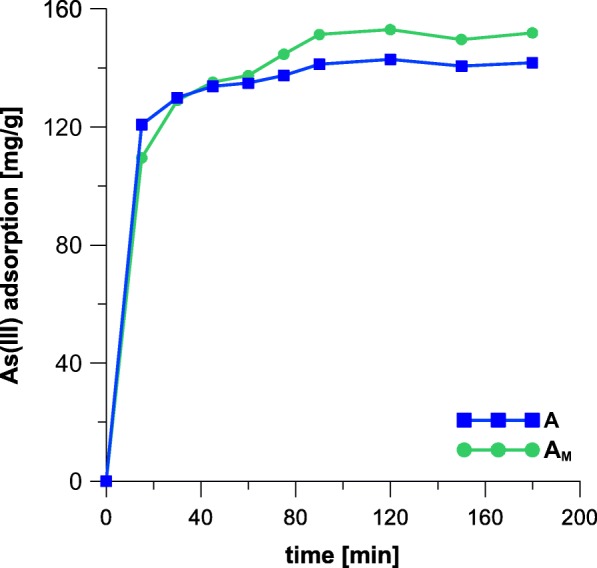
Kinetics of As adsorption onto akaganeite (A) and modified akaganeite (A_M_)

As shown in Fig. 5, the As adsorption onto iron oxides is considerably fast during the initial period of the process. Adsorption equilibrium was reached after 90 min. It is worth mentioning that the modification of akaganeite with HDTMA improved its sorption capacity.

Within the present studies, kinetics of the adsorption of As was modeled using pseudo-second-order kinetic model (PSO) in its linear form (Eq. 1). The model provides good correlation of the experimental data in many cases (Mohan and Pittman 2007; Mostafa et al. 2011; Sarı and Tuzen 2009). The values of *k*_2_ and *q*_*m*_ were calculated from the slope and intercept of the *t*/*q*_*t*_ versus *t* plots, respectively. Additionally, initial adsorption rate (mg g^−1^ min^−1^) defined as $h={k}_2{q}_m^2$ was calculated. The resultant values are summarized in Table 2. Values of the correlation coefficients (*R*^2^) are close to 1, indicating that the model complies with the experimental adsorption capacity; thus, the model is selected properly for the present studies. The calculated adsorption capacities (*q*_*m*_) are similar to the experimental ones (*q*_exp_); however, pseudo-second-order rate constant (*k*_2_) is over twice lower in the case of the modified iron oxide (A_M_), which is probably directly correlated with the significant difference between initial adsorption rates (*h*; Table 2). This can indicate that the internal surface area (Table 1) of the sample A_M_, which is developed to a lesser extent than in the case of the unmodified mineral A, can cause diffusional limitations experienced by the adsorbate. For this reason, an intraparticle pore diffusion kinetic model (IPD) was also applied to the present results.

**Table 2:** PSO kinetic model parameters calculated for the adsorption of As

Sample	*q*_exp_^a^	*q*_*m*_^b^	*k*_2_^c^	*h* ^d^	*R* ^2^
A	142.8	144.7	2.07 × 10^−3^	43.3	0.9997
A_M_	153.0	158.3	0.92 × 10^−3^	23.0	0.9990

*A* akaganeite, *A*_*M*_ modified akaganeite

^a^Experimental adsorption capacity (mg g^−1^)

^b^Adsorption capacity received form PSO equation (Eq. 1) (mg g^−1^)

^c^PSO rate constant (g mg^−1^ min^−1^)

^d^Initial adsorption rate (mg g^−1^ min^−1^)

**Fig. 6:**
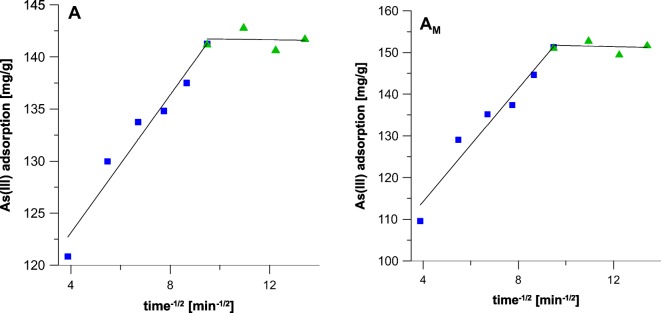
Intraparticle pore diffusion kinetic model applied for adsorption of As on (A) akaganeite and (A_M_) modified iron oxide

Figure 6 displays plots of IPD kinetic model applied for the adsorption of As on the iron oxides, while Table 3 provides parameters calculated using thereof.

**Table 3:** Parameters of IPD kinetic model

Sample^a^	First stage		
	*k*_*d*_^a^	Θ^b^	*R* ^2^
A	3.1	109.9	0.95
A_M_	6.8	87.1	0.95

^a^IPD rate constant (mg (g^−1^ min^−1/2^))

^b^Measure of boundary layer thickness (mg g^−1^)

As can be seen in Fig. 6, the adsorption of As on both adsorbents is a two-stage process. The rates of the first stages are assigned to the macropore and mesopore diffusion (Fierro et al. 2008). Contrary to the PSO model, the IPD adsorption rate in the initial stage of adsorption is over twice greater in the case of the modified sample (*k*_*d*_; Table 3). The phenomenon is surprising, as the film diffusion should be much easier in the case of pure akaganeite which internal surface area is much more developed. However, the PSO kinetics considers initial state of the process as a whole, from its beginning to the end, while IPD model is focused on film diffusion, not taking into account adsorption at time equal to 0.

The values collected within Table 3 indicate that adsorption of arsenic on the both samples reveal a complex character. As already reported in the literature (Deliyanni et al. 2006), the adsorption on iron oxides involves an electron transfer from Fe and O atoms; thus, it reveals chemical character. However, as displayed in Table 3, the value of Θ which is the measure of a theoretical boundary layer thickness is smaller in the case of the modified akaganeite. This could mean that HDTMA makes the mineral surface more accessible for the adsorbate. Hence, the greater efficiency of the sample A_M_ is observed.

### Equilibrium Studies

**Fig. 7:**
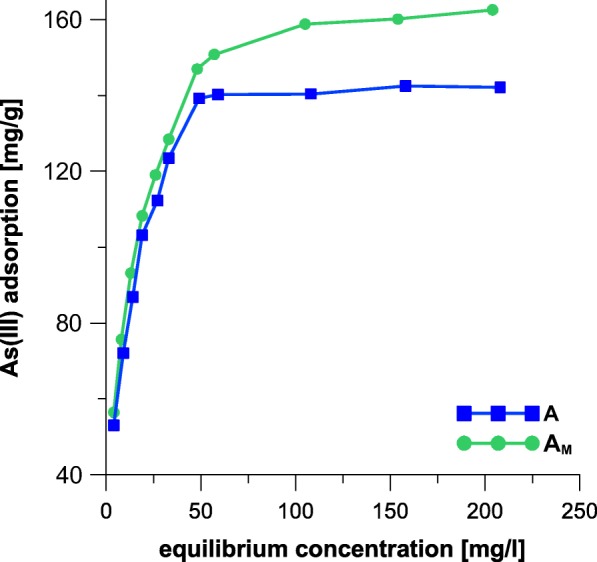
Adsorption of As on akaganeite (A) and modified akaganeite (A_M_)

The obtained As adsorption isotherms expressed as adsorption capacity in function of equilibrium concentration are displayed in Fig. 7.

The experimental data were recalculated using with Langmuir equation (Eq. 3), as predictably most suitable model in the present studies (Deliyanni et al. 2003; Deliyanni and Matis 2005; Mostafa et al. 2011; Pirilä et al. 2011). Values of *Q*_0_ and *b* were calculated from the slope and intercept of the linear plot of *c*_eq_/*q*_*e*_ versus *c*_eq_, respectively. The Langmuir constants and parameters (Eq. 3) are listed in Table 4.

**Table 4:** Parameters and *R*^2^ values for the Langmuir model

Sample	*Q*_0_^a^	*b* ^b^	*R* ^2^	*R*_*L*_ (initial concentration: 4 mg L^−1^)	*R*_*L*_ (initial concentration: 250 mg L^−1^)
A	148.7	0.1365	1.00	0.65	0.03
A_M_	170.9	0.1033	1.00	0.71	0.04

*A* akaganeite, *A*_*M*_ modified akaganeite

^a^Adsorption capacity (mg g^−1^)

^b^Langmuir adsorption constant (L mg^−1^)

The essential characteristic of the Langmuir isotherm can be represented by the parameter *R*_*L*_ which demonstrates the shape of the isotherm. If *R*_*L*_ is higher than 1, the adsorption is unfavorable. If *R*_*L*_ is equal to 1, the adsorption is linear. If the parameter *R*_*L*_ has value lying in the range of 0 to 1, the adsorption can be described as favorable. If *R*_*L*_ is equal to 0, the adsorption is irreversible (Öztürk and Kavak 2005). As displayed in Table 2, the values of *R*_*L*_ are very small but lying in the range of 0 to 1; therefore, the adsorption of As onto akaganeite-type adsorbents is a favorable process. The *R*_*L*_ values vary in the range, respectively, 0.03–0.65 (A) and 0.04–0.71 (A_M_). These values decrease when initial concentration of a solution increases.

The *Q*_0_ values determined for the samples A and A_M_ are comparable (Table 2) despite ten-fold difference between the BET surface area (Table 1) of these adsorbents. This observation confirms that the efficiency of adsorption is not linked with porous structure of the obtained samples. As far as the process complied with Langmuir model (high values of *R*^2^; Table 2), a conclusion that the adsorbate creates monolayer on the surface of adsorbent, thus it reveals chemical character, can be made. The problem is that As species are neutral at pH between 6 and 9 (Yazdi and Darban), while the present studies were carried out at pH 7. If the investigated adsorption mechanism reveals chemical character, the initial As(III) must had been oxidized to As(V) at pH 7. The phenomenon is often reported in literature data, as the As(III) is unstable at higher pH (Yazdi and Darban; Takeno 2005). At pH above 6, the As(III) species oxidizes to As(V), appearing in the ionic form of HAsO_4_^2−^ (Takeno 2005). However, as indicated before (Deliyanni et al. 2006), the mechanism of arsenic adsorption does not involve ion exchange on the quaternary ammonium group introduced together with HDTMA, but on the surface of iron oxide itself. Therefore, the reason of the greater adsorption capacity registered for the sample A_M_ should be found in the parameters of the applied IPD kinetic model as described above. The value of Θ (Table 3), which is the measure of theoretical boundary layer thickness, is greater in the case of the modified iron oxide. The phenomenon is directly attributed to the impact of the HDTMA introduced into adsorbent, as the quaternary ammonium group affects the charge on Fe- and O-containing functional groups present in the akaganeite. This further makes the diffusion of arsenic within the adsorbent easier (Deliyanni et al. 2006). Within the present studies, we postulate that the phenomenon makes the active sites more accessible, thus improving adsorption capacity of the so-prepared adsorbent.

### Presented Iron Oxides Versus Adsorbents Reported in Literature

Table 5 displays comparison of the adsorption capacities of arsenic adsorbents investigated in the present studies to other ones reported in the worldwide literature.

**Table 5:** Synthetic iron oxides compared to other adsorbents of arsenic

Adsorbent	Pollutants	*Q*_max_ (mg g^−1^)	References
Mesoporous γ-alumina	As(V)	19.1	Tchieda et al. (2016)
Zeolite (H24)	As(V)	35.8	Chutia et al. (2009)
Macrofungus biomass	As(V) As(III)	59.6 51.9	Sarı and Tuzen (2009)
Biogenic schwertmannite	As(III)	113.9	Liao et al. (2011)
Soot	As(V) As(III)	30.5 29.9	Pattanayak et al. (2000)
Fly ash	As(V)	30.0	Diamadopoulos et al. (1993)
Fly ash	As(III)	74.4	Polowczyk et al. (2010)
Iron oxide coated cement	As(III)	0.3	Kundu and Gupta (2007)
Akaganeite	As(V)	134.1	Deliyanni et al. (2003)
Akaganeite	As(III)	148.7	Present studies
Akaganeite modified	As(V)	170.9	

Based on the already reported solutions, the adsorption of arsenic on low-cost adsorbents varies from 0.3 to 134 mg g^−1^. As can be seen in Table 5, application of waste materials can be attractive, as adsorption of As(III) and As(V) is comparable or even grater to the corresponding processes carried out on zeolites and alumina-based adsorbents. Based on the data collected in the literature, as well as these presented in the present work, it can be concluded that application of mineral iron-oxide adsorbents reveals superiority of such a solution. As far as removal of arsenic on the pure akaganeite is comparable to the capacity of similar mineral reported by Deliyanni (Deliyanni et al. 2003), the modification thereof indeed improved adsorption of As. Removal of arsenic in fixed conditions for the sample A_M_ was approximately 30 mg g^−1^ greater than these received for unmodified iron oxides (Table 5). It must be also emphasized that when an excess of arsenic is applied during the process, the maximum adsorption capacity on the sample A_M_ can be further increased to 200 mg g^−1^ (see Fig. 3), making the reported modification of akaganeite a very attractive solution for enhancing processes of As removal.

## Conclusion

Within the present studies, the As adsorption on akaganeite (A) and akaganeite modified with HDTMA was assessed. Based on the obtained data, the following conclusions can be made:

- Comparing to literature data, both the akaganeite (A) and the surfactant-modified akaganeite (A_M_) are very efficient adsorbents for As removal.
- Modification of akaganeite resulted in significant decrease of its specific surface area, indicating that the HDTMA was indeed immobilized within structure of the mineral.
- The akaganeite modified with HDTMA reveals greater adsorption capacity, thus the ability to remove As.
- Although the modification of akaganeite did improve its adsorption capacity, the present studies also revealed that As loading capacity is not dependent on the porous structure of these materials.
- Introduction of HDTMA into the iron oxide decreases the thickness of theoretical boundary layer; hence, its positive effect on adsorption capacity is observed.
- Because the As adsorption complies with Langmuir and PSO models and no linkage with specific surface area was found, it can be stated that the process reveals a chemical character.
- Based on the IPD kinetic model, it can be stated that the thickness of the theoretical boundary layer is smaller in the case of the modified mineral; thus, the diffusion is enhanced.
- Adsorption of As on iron oxides should be carried out at pH around 7.5 as the speciation of As favors chemical adsorption.
